# Supplementary material for: Seminoma and Embryonal Carcinoma Footprints Identified by Analysis of Integrated Genome-Wide Epigenetic and Expression Profiles of Germ Cell Cancer Cell Lines
Source: PLoS One. 2014 Jun 2;9(6):e98330. doi: 10.1371/journal.pone.0098330 (PMC4041891; doi:10.1371/journal.pone.0098330)
Supplement: File S1 — ZIP file containing DMRforPairs output for significant regions. Please start from the html files. (ZIP) [file pone.0098330.s008.zip › figures/39388.pdf]

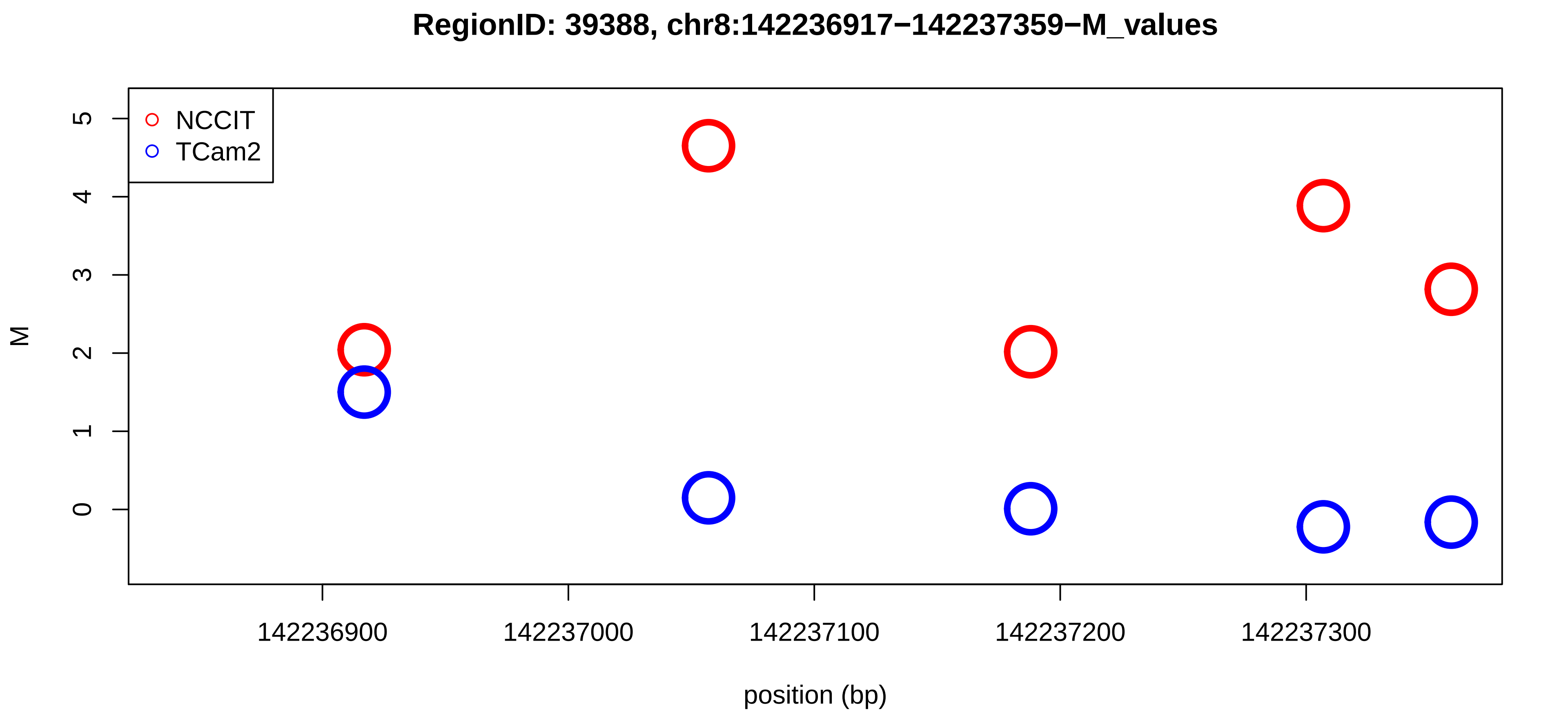
RegionID: 39388, chr8:142236917−142237359−M_values
NCCIT
TCam2
M
0
1
2
3
4
5
142236900
142237000
142237100
142237200
142237300
position (bp)

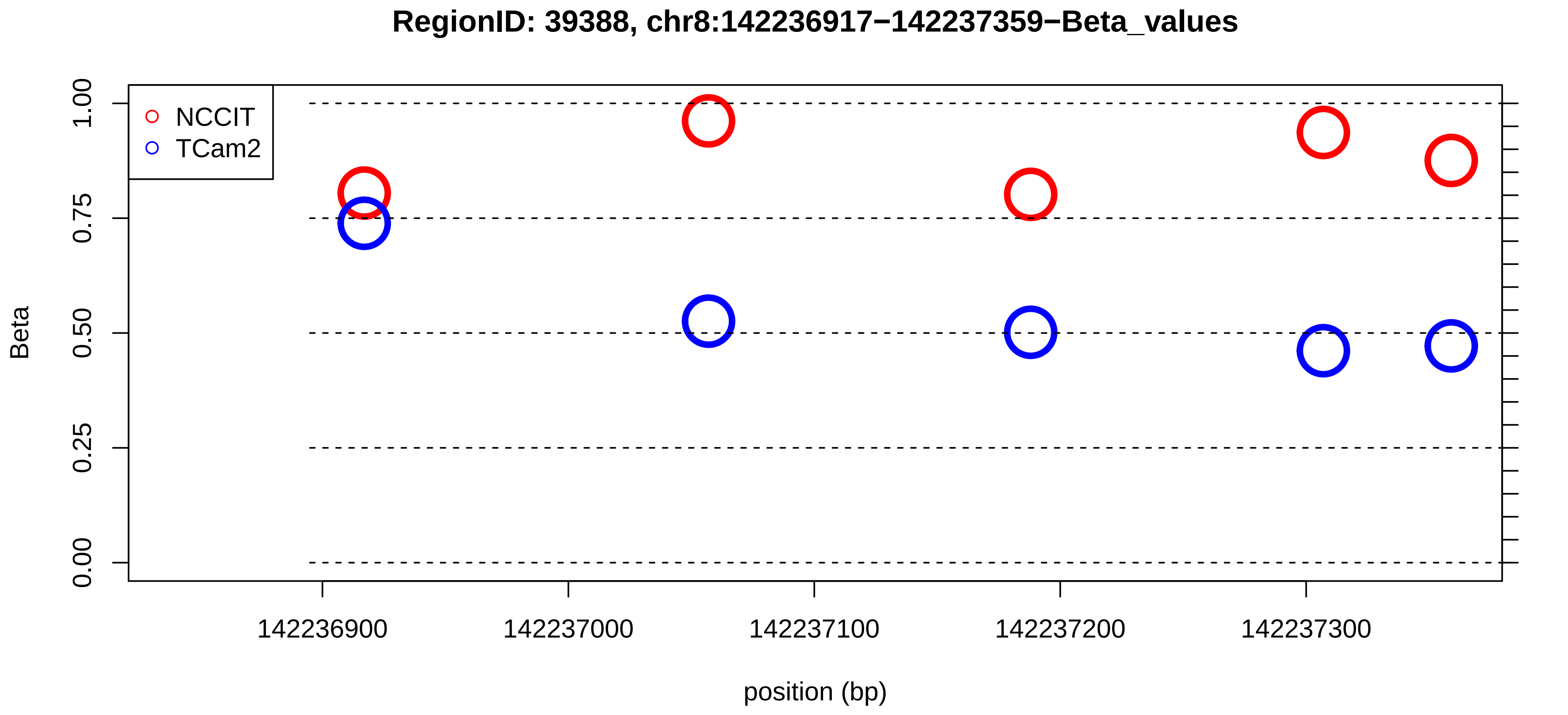
RegionID: 39388, chr8:142236917−142237359−Beta_values
NCCIT
TCam2
Beta
0.00
0.25
0.50
0.75
1.00
142236900
142237000
142237100
142237200
142237300
position (bp)
